# *Aspergillus* nodules; another presentation of Chronic Pulmonary Aspergillosis

**DOI:** 10.1186/s12890-016-0276-3

**Published:** 2016-08-18

**Authors:** Eavan G. Muldoon, Anna Sharman, Iain Page, Paul Bishop, David W. Denning

**Affiliations:** 1 The National Aspergillosis Centre, University Hospital of South Manchester, 2nd floor ERC, Southmoor Road, Wythenshawe, M23 9LT UK; 2 The Department of Radiology, University Hospital of South Manchester, Southmoor Road, Wythenshawe, M23 9LT UK; 3 The Department of Histopathology, University Hospital of South Manchester, Southmoor Road, Wythenshawe, M23 9LT UK; 4 The University of Manchester and the Manchester Academic Health Service Centre, University Hospital of South Manchester, Southmoor Road, Wythenshawe, M23 9LT UK

**Keywords:** Aspergillus, Pulmonary nodule, Fungal infection of lung, Chronic pulmonary aspergillosis

## Abstract

**Background:**

There are a number of different manifestations of pulmonary aspergillosis. This study aims to review the radiology, presentation, and histological features of lung nodules caused by *Aspergillus spp*.

**Methods:**

Patients were identified from a cohort attending our specialist Chronic Pulmonary Aspergillosis clinic. Patients with cavitating lung lesions, with or without fibrosis and those with aspergillomas or a diagnosis of invasive aspergillosis were excluded. Demographic, laboratory, and clinical data and radiologic findings were recorded.

**Results:**

Thirty-three patients with pulmonary nodules and diagnostic features of aspergillosis (histology and/or laboratory findings) were identified. Eighteen (54.5 %) were male, mean age 58 years (range 27–80 years). 19 (57.6 %) were former or current smokers. The median Charleston co-morbidity index was 3 (range 0–7). All complained of a least one of; dyspnoea, cough, haemoptysis, or weight loss. None reported fever. Ten patients (31 %) did not have an elevated *Aspergillus* IgG, and only 4 patients had elevated *Aspergillus* precipitins. Twelve patients (36 %) had a single nodule, six patients (18 %) had between 2 and 5 nodules, 2 (6 %) between 6 and 10 nodules and 13 (39 %) had more than 10 nodules. The mean size of the nodules was 21 mm, with a maximum size ranging between 5–50 mm. No nodules had cavitation radiographically. The upper lobes were most commonly involved. Histology was available for 18 patients and showed evidence of granulation tissue, fibrosis, and visualisation of fungal hyphae.

**Conclusion:**

Pulmonary nodules are a less common manifestation of aspergillosis in immunocompetent patients. Distinguishing these nodules from other lung pathology may be difficult on CT findings alone.

## Background

There are a number of manifestations of pulmonary aspergillosis [1]. *Aspergillus spp.* are ubiquitous in the environment and exposure to conidia is common. However, only a minority of people develop clinical disease, and this is often determined by host characteristics, e.g. immune compromise, genetic predisposition, underlying lung pathology, and prior pulmonary infection such as tuberculosis (TB). Classically chronic pulmonary aspergillosis (CPA) in immunocompetent patients presents as a saprophytic infection in a pre-existing cavity, often following an infection such as TB or prior lung surgery. There are a number of recognised manifestations of CPA; subacute invasive pulmonary aspergillosis (SAIA) [which may be referred to as chronic necrotising pulmonary aspergillosis (CNPA)], chronic cavitary pulmonary aspergillosis (CCPA) and chronic fibrosing pulmonary aspergillosis (CFPA) [2]. Subacute IPA occurs in the setting of some degree of immune compromise, and may present with nodules, consolidation and or cavitation on chest imaging, and a more rapidly progressive clinical course. CCPA presents with single or multiple cavities, with or without aspergilloma(s), and CFPA has this appearance with the additive features of pulmonary fibrosis, which may be progressive and destructive.

Estimates of the incidence and prevalence of CPA are difficult; however the global burden of disease is increasingly being recognised [3, 4]. For example, the proportion of patients with TB as an underlying risk factor for the development of CPA will vary depending on geographical location [5]. In 2011, CPA was estimated to affect 3600 patients in the UK, based on estimates of prior TB, and diagnoses of sarcoidosis [6], and 1.2 million worldwide after TB [3] and 72,000 complicating fibrocystic pulmonary sarcoidosis [4].

There is a paucity of knowledge on CPA which presents as single or multiple nodule(s) without cavitation in immune competent hosts. The published literature is limited to case reports and small case series. Often, in these cases the diagnosis is made following removal or biopsy of the nodule(s) which is presumed to be malignant [7, 8]. In the largest case series from Korea, eleven patients with solitary pulmonary nodules were reviewed [9]. Three of the eleven patients had some evidence of cavitation on CT imaging, and all had histologically proven *Aspergillus* infection. In a second Korean series, seven patients were identified with biopsy proven *Aspergillus* disease, in the absence of immunosuppression or underlying lung disease [10]. Unfortunately in neither series was a correlation made with *Aspergillus* IgG (precipitins), which is a cornerstone of the diagnosis of CPA [2].

The purpose of this study is to review the clinical characteristics, histological and radiological features of pulmonary nodules caused by *Aspergillus spp*.

## Methods

Patients attending our specialist CPA clinic in the National Aspergillosis Centre (NAC) with nodular *Aspergillus* disease were identified. The NAC is nationally commissioned to provide specialist care for patients with chronic pulmonary aspergillosis in the UK. There are currently approximately 350 patients in follow up care of the NAC with CPA, and approximately 100 new patients referred annually. Patients were identified by one of two methods. First, patients with pulmonary nodules on chest imaging at presentation, and features consistent with a diagnosis of aspergillosis (i.e. biopsy proven disease and/or positive *Aspergillus* serology and/or *Aspergillus spp* isolated form respiratory secretions) were prospectively recorded. Second, additional case finding was performed by the retrospective review of patient correspondence and review of histopathology records. A rounded opacity, well or poorly defined, measuring up to 3 cm in diameter was defined as a nodule as per the Fleischner Society: Glossary of Terms for Thoracic Imaging [11]. Patients with aspergillomas and those with cavitating lung lesions, with or without fibrosis were excluded. Patients with a diagnosis of invasive aspergillosis were also excluded. Demographic data, details of the clinical presentation, laboratory data and radiologic findings were recorded on each patient. All radiology was reviewed by a consultant radiologist (AS) for accuracy. The ImmunoCap™ assay (Phadia, Uppsala, Sweden) was used to measure *A. fumigatus* IgG and the Microgen antigens and counterimmunoelctrophoresis (MIcrogen, Camberley, Surrey, UK) for Aspergillus precipitins. Serum mannose binding lectin (MBL) concentrations were measured by ELISA (MBL Oligomer ELISA Kit, BioPorto Diagnostics, DK), upper and lower reported detection limit of 4.00 and 0.05 mg/L respectively. For culture, sputum was digested with Sputasol® (ratio 1:1), vortexed, and 10 μL-streaked on two Sabouraud dextrose agar plates [12] and incubated at 30 °C and 37 °C for 7 days, and on bacterial media. For quantitative PCR, the MycXtra kit (Myconostica, Cambridge, UK) was used for DNA extraction using 0.5–3 mL of sample. DNA was eluted in 40 μL and 10 μL used for quantitative PCR. The MycAssay Aspergillus kit (Myconostica) was used following the manufacturer’s instructions; a crossing threshold (Ct) of >38 was negative, Ct from 36–38 a weak positive and <36 was interpreted as a strong positive [13]. The data were collected in Microsoft excel, and data analysis performed using SPSS version 20. This report is a retrospective evaluation of all patients who were managed with *Aspergillus* nodules, and as such is exempt from ethical review or patient consent.

## Results

Thirty three patients with lung nodules and features diagnostic of CPA (histology and/or laboratory findings) were identified. Ten patients had proven disease, and the remainder deemed probable disease, based on serology and culture results (Table 1). Eighteen (54.5 %) of patients were male, the mean age was 58 years (range 27–80 years). Nineteen (57.6 %) were current or ex-smokers, in 9 (27.3 %) smoking history was not documented. The median Charleston co-morbidity index was 3 (range 0–7). On presentation all patients complained of a least one of the following symptoms; dyspnoea, cough, haemoptysis, or weight loss. Twenty nine patients (88 %) reported cough, 23 (70 %) dyspnoea, 11 (33 %) described weight loss, and 5 (15 %) haemoptysis. No patients reported a history of fever.

**Table 1:** Characteristic of patients diagnosed with Aspergillus nodule(s)

Patient	Number of nodules	Lobes of lung involved	Min size (mm)	Max size (mm)	Lymphadenopathy	Visible on concurrent CXR	Symptoms	Aspergillus IgG	Sputum culture	Aspergillus PCR	Tissue Specimen	Results
Aspergillus Nodules												
1	1	LLL		11	N	Y	None	76		n/a	lung	fibrosis, fungal hyphae
2	2	Upper lobes bilaterally	4	10	n	y	None	68		Negative	lung	granuloma, necrosis, fungal hyphae
3	1	LUL		16	n	y	Dyspnoea, cough, weight loss	14		Negative	lung	Inflammation, fungal hyphae
4	2	RUL		7	n	y	Cough, weight loss	40		Negative	Lung	Inflammation granulomatous
5	1	RUL		16	n	y	Dyspnoea, weight loss	101		n/a	lung	COP, fungal hyphae
6	1	RUL		12	n	y	Dyspnoea, cough, haemoptysis	N/A	A. fumigatus	n/a	BAL	inflammatory infiltrate fungal hyphae
7	1	LUL		22	n	y	Dyspnoea, cough	22		N/A	lung	fibrosis, granulomata, necrosis, fungal hyphae
8	1	RUL		22	n	y	Dyspnoea, cough, haemoptysis	86		Negative	lung	Inflammation, fungal hyphae
9	3	Upper lobes bilaterally	6	27	n	y	Dyspnoea, cough, weight loss	54	A. fumigatus	Negative	lung	fungal hyphae, necrosis
10	1	LLL		25	could not visualise	y	Dyspnoea, cough	23	A. fumigatus	Positive	Lung	Inflammation granulomatous
11	2	LUL	9	35	n	y	Dyspnoea, cough	32		Weak positive	Lung	Inflammation granulomatous
Probable Aspergillus Nodules												
12	4	All lobes	2	38	y	y	Dyspnoea, cough, haemoptysis	185		Positive		
13	2	Upper lobes bilaterally	3	9	n	N/A	Cough	87		Negative		
14	4	All lobes except RUL	2	16	n	y	Dyspnoea, cough	49		n/a	BAL	benign cells, polymorphs
15	2	Upper lobes bilaterally	7	12	y	y	Dyspnoea, cough	68		Negative		
16	4	all lobes except RML	5	16	n	Y	Dyspnoea, cough	65	A. nidulans, A. niger	Negative		
17	1	LUL		14	n	Y	Dyspnoea, cough, weight loss	152		N/A	BAL	benign cells, polymorphs
18	1	RUL		10	n	y	Cough, weight loss	18		n/a		
19	4	All lobes	2	31	Y	Y	Dyspnoea, cough, haemoptysis, weight loss	52	A. fumigatus	Weak positive	lung	haemosiderin deposition
20	4	Upper lobes bilaterally	1	13	y	y	Dyspnoea, cough	190	A. fumigatus	Weak positive	lung	inflammatory debris
21	4	All lobes except RUL	1	18	n	y	Dyspnoea, cough	115	A fumigatus	Positive		
22	1	LUL		16	n	y	Dyspnoea, cough, weight loss	19	A nidulans, A.fumigatus	Weak positive		`
23	4	All lobes	1	16	y	n	Dyspnoea, cough	75		N/A	Lung	Emphysema bullous
24	1	RUL		20	n	y	Cough	12	A. fumigatuis	N/A		
25	4	All lobes	1	20	y	Y	Cough	170		Negative		
26	4	All lobes	5	34	n		Dyspnoea, cough, weight loss	104		Weak positive		
27	4	All lobes	1	5	n	y	Dyspnoea, cough	42	A. fumigatus	Positive		
28	4	RUL	1	27	n	y	Cough, haemoptysis	82		Positive		
29	4	all except RML, RLL	1	28	N	Y	Dyspnoea	42.5		N/A		
30	3	LUL	5	37	n	y	Cough	106		Negative		
31	1	RUL		29	n	y	Cough	18		Negative		
32	4	All lobes	6	32	n	y	Dyspnoea, cough, weight loss	10		N/A		
33	2	LUL	17	50	n	N/A	Dyspnoea, cough, weight loss	108		Negative		

### Radiological features

**Fig. 1:**
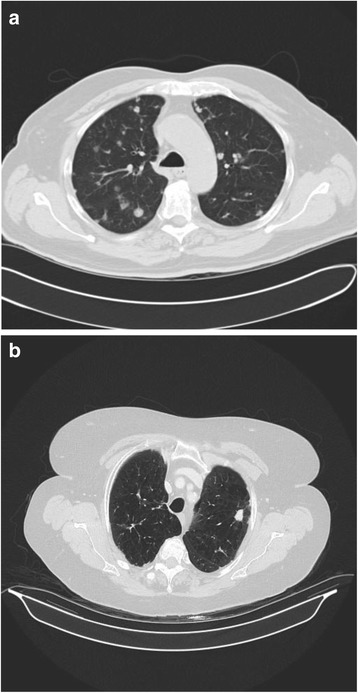
Different patterns of Aspergillus nodule disease. A showing multiple nodules, B a single pulmonary nodule on background of emphysematous lungs

**Fig. 2:**
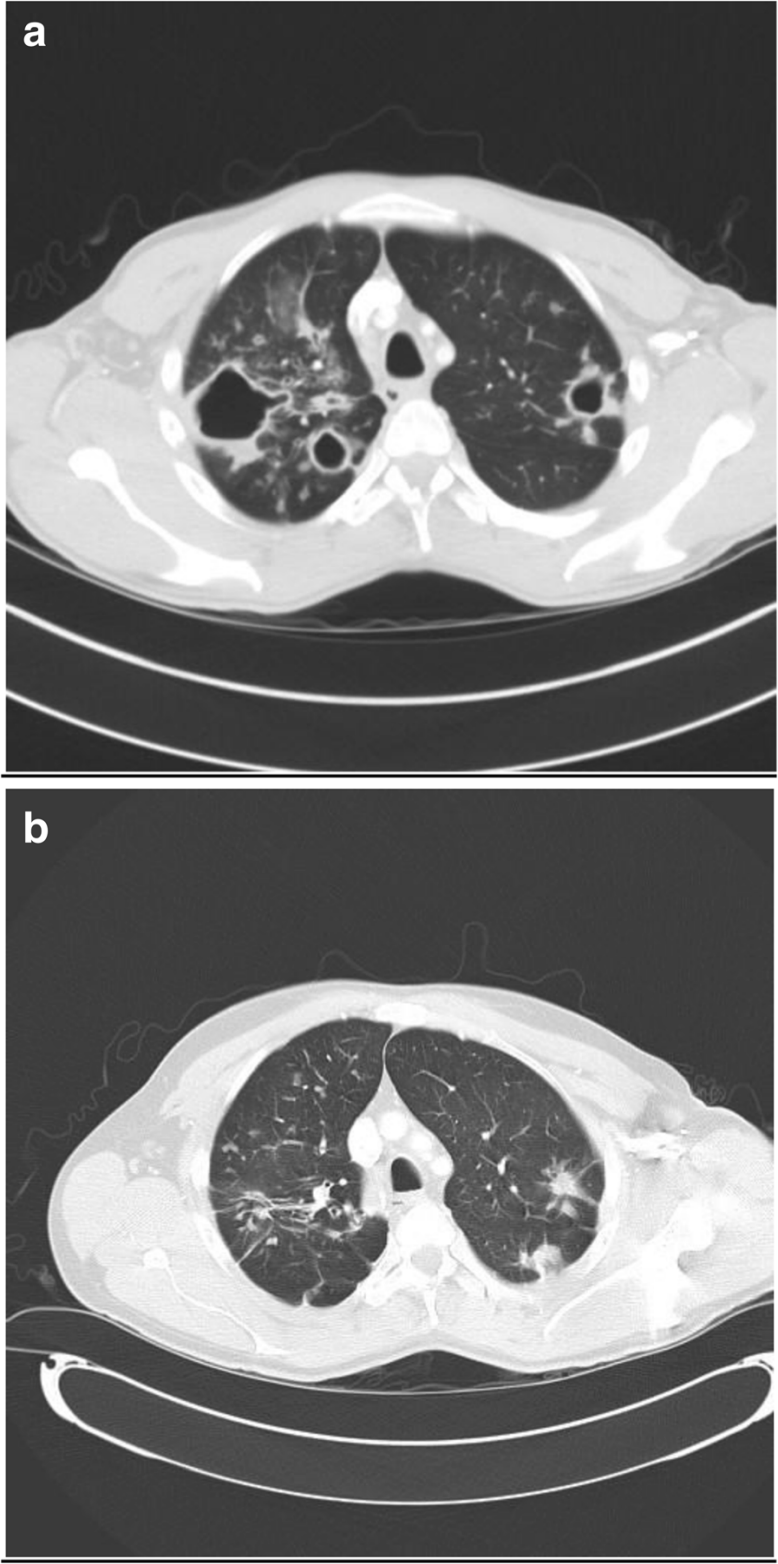
CT imaging showing cavitating disease initially, which developed into nodular disease

All patients had computer tomography (CT) performed. Twenty patients (60 %) had upper lobe disease alone, with either unilateral or bilateral involvement. In seven patients (6 %) all lobes were involved, the remaining patients had variable patterns of lobar involvement. In twelve patients (36 %) a single nodule was present, six (18 %) patients had between 2 and 5 nodules, 2 (6 %) had between 6 and 10 nodules and 13 (39 %) patients more than 10 nodules. The maximum nodule size ranged between 5–50 mm, mean 21 mm. Associated lymphadenopathy was present in six patients (18 %). Thirty patients had a plain chest film performed concurrently with the CT imaging, in 29/30 (97 %) the nodule was visible on plain film. Eight patients (24 %) had undergone positron emission tomography (PET) and in all cases the fluorodeoxyglucose (FDG) uptake was low to moderate (SVUmax <5.4). Twenty three patients (70 %) had a solid mass on CT imaging (Fig. 1), while the remaining patients had a mixed pattern of disease. Eleven patients had findings consistent with emphysema on CT imaging. One patient initially had a cavitating lesion which became solid on repeat imaging (Fig. 2). Only one patient, with multiple nodules, had evidence of calcification within some nodules.

### Laboratory parameters

*Aspergillus* IgG antibody results were available for 32 patients. In ten patients (31 %) the *Aspergillus* IgG was within normal limits (i.e. ≤40 mg/L), including four patients with a result between 20 mg/L and 40 mg/L. *Aspergillus* precipitins was positive in 4/32 (12.5 %). Eight patients (24 %) had lymphopenia (lymphocyte count <1.5 × 10^9^/L). Twenty nine patients had MBL measured, and 11/29 (38 %) were deficient (<1.0 mg/L). Twenty nine patients submitted sputum samples for analysis. Nine of the 32 patients (31 %) isolated an *A. fumigatus* from their sputum sample, one of whom also had *A. nidulans* isolated from their sputum. One patient had *A. nidulans* and *A. niger* isolated from his sputum samples. Sputum samples also yielded a number of bacterial organisms including *S. aureus, H. influenzae, H. parainfluenzae, M. catarrhalis, S. marcescens, E. coli, K. pneumoniae, S. maltophilia, and P. aeruginosa*. Seven patients had sputum samples which did not yield any growth of bacteria or fungi. Twenty two patients submitted sputum for *Aspergillus* PCR analysis, 10/22 (45 %) were positive. In four cases (4/14) *Aspergillus* DNA was detected by PCR but there was no growth of *Aspergillus spp.* by culture.

### Histology

**Fig. 3:**
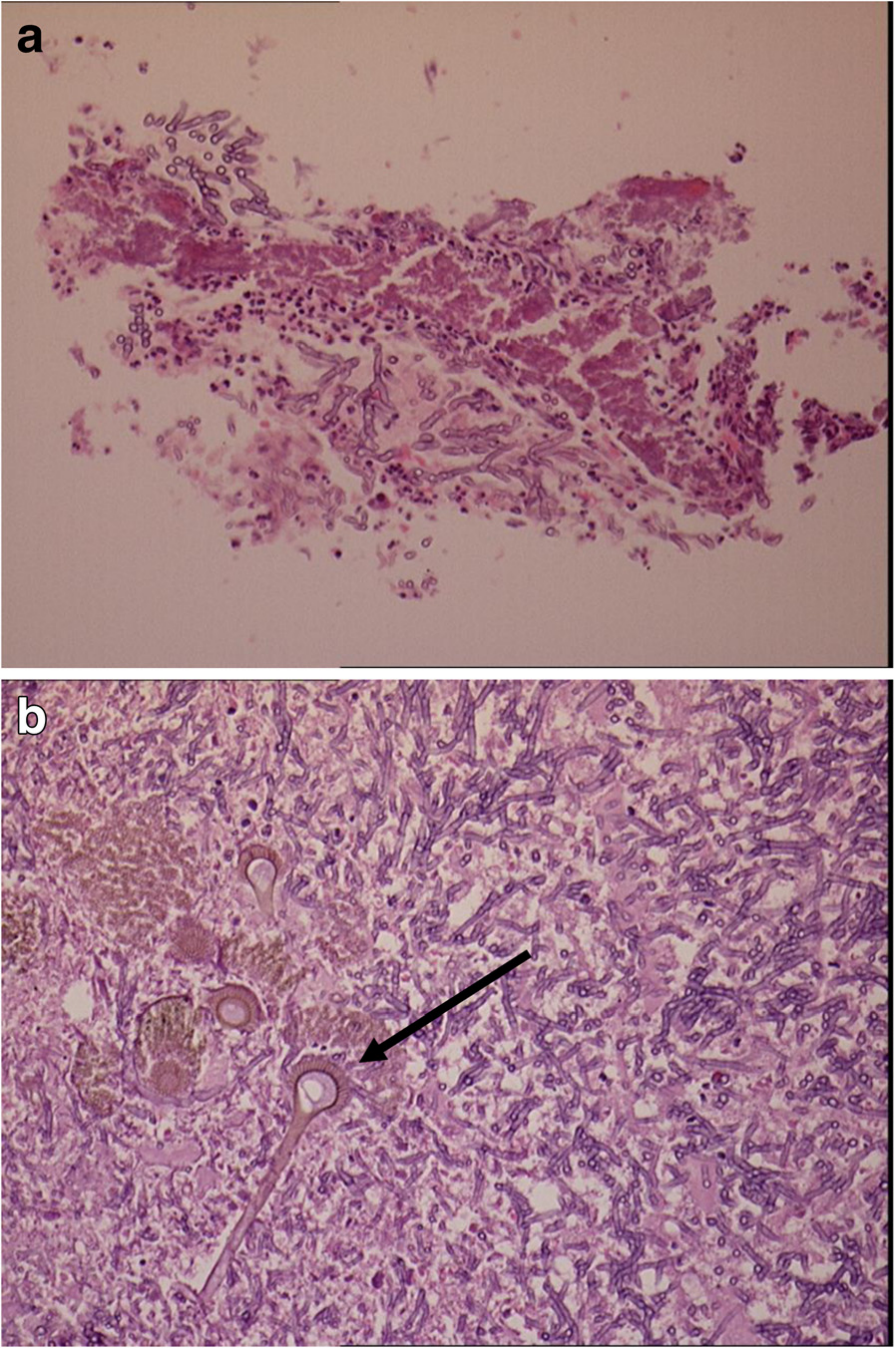
Biopsy demonstrating inflammation and necrosis and fungal hyphae (*low power*) and fungal conidia (*arrow*) on high power

Histology was available on sixteen patients. Thirteen (81 %) had undergone lung biopsy, and the remainder had bronchoalveolar lavage (BAL) fluid analysed. Of those who had undergone lung biopsy, in 7/13 (54 %) fungal hyphae were visualised. Granulomatous inflammation and/ or necrosis was seen in the remaining patients histology (Table 1). Of the three patients who had BAL washings available for analysis, one had fungal hyphae visualised in bronchial washings. Some had fruiting bodies (conidiophores with conidia) of *Aspergillus* identified (Fig. 3), suggesting that the original infection with *Aspergillus* occurred in an airspace or on an epithelial surface, and subsequently was filled in with inflammatory cells and *Aspergillus* hyphae.

### Surgery

Nine patients underwent surgical resection of the lung nodule. One patient had recurrent disease identified on CT four years post operatively.

## Discussion

In this, the largest published series of *Aspergillus* nodules to date, the characteristics of 33 patients were reviewed. These patients represent less than 10 % of the cohort of patients with CPA cared for in the National Aspergillosis Centre. However, this may be an under representation of this presentation of CPA, as cases may not be recognised, have negative *Aspergillus* IgG or precipitins, and/or not undergo biopsy to secure their diagnosis. However, recognition of nodules on CT scanning of the thorax is becoming more common, as screening for lung cancer is more frequently undertaken. Many nodules identified on such screening scans are removed or biopsied and do not reveal malignancy. *Aspergillus* nodules are one such benign entity.

In those unable to undergo biopsy or resection because of poor respiratory reserve and a risk of pneumothorax, empirical radiotherapy is sometimes given for a ‘PET positive’ suspicious lesion. We have seen at least 2 patients with chronic pulmonary aspergillosis in the area of radiotherapy, which we suspect, but cannot prove, had an *Aspergillus* nodule that was irradiated. We would therefore encourage clinical oncologists to consider the possibility of an *Aspergillus* nodule before embarking on lung irradiation. The response to radiotherapy may not be entirely problematic however, as illustrated by a small series of CPA patients explicitly treated with radiotherapy [14].

A number of other infections may also present with pulmonary nodules, which may be difficult to distinguish on radiological features alone (Table 2). The relative frequency of the differential diagnoses varies substantially by geography. In endemic areas, other fungal infections can present with persistent pulmonary nodules of masses in apparently immunocompetent persons. The appearance of such fungal infections mimics malignancy and diagnosis is often confirmed on biopsy. At one centre in Texas, USA 17 of 2,098 (0.6 %) persons presenting with pulmonary nodule were ultimately diagnosed with histoplasmosis, cryptococcosis or coccidiomycosis rather than malignancy [15]. Another case series describes 27 cases of fungal lung infection presenting with persistent lung nodule or mass at 2 centres in Texas USA and Sao Paulo Brazil respectively [16]. All cases were referred for investigation of suspected malignancy. Diagnoses included histoplasmosis (26 %), coccidioidomycosis (22 %), cryptococcosis (22 %), aspergillosis (15 %), blastomycosis (7 %), mucormycosis (4 %) and paracoccidioidomycosis (4 %). Fourteen (52 %) of patients had a past history of treated malignancy and 15 (56 %) were symptomatic at presentation. Thirteen (48 %) of patients had cough, 7 (26 %) had chest pain and 7 (26 %) weight loss. Increased PET avidity was noted in all patients and all patients demonstrated radiological improvement or resolution with appropriate antifungal therapy.

**Table 2:** Infectious differential diagnosis of pulmonary nodules

Cause of nodule/disease	Underlying disease(s), geography	CT characteristics	Evolution
*Aspergillus* nodule	Emphysema, asthma taking corticosteroids, smoker. Not immunocompromised. Global	Single or multiple nodules. May affect any lobe, although upper lobes most common. Unlikely to be calcified	Slow to change. May cavitate over many months.
Coccidioidal nodule	None. Visit to, or inhabitant of, endemic area.	Usually single, upper lobes. Occasionally calcified.	Static over months or years.
*Histoplasma* nodule	None. Visit to, or inhabitant of, endemic area. May report specific exposure e.g bat cave	Single or multiple. Often calcified.	Static over months or years.
Nontuberculous mycobacterial nodule	Emphysema, corticosteroids, bronchiectasis. Global	Single or multiple. May be calcified. >5 mm diameter.	Progressive
*Pneumocystis jirovecii*	Usually immunocompromised patients, HIV, steroids etc.	Single/multiple	
	Very rare cause of nodules in immunocompetent host. Global		
*Nocardia spp.*	May mimic TB	Single or multiple	
	Up to 1/3 cases occur in immunocompetent hosts. Global		
Dirofilariasis	None. Mosquito borne zoonosis, travel to South East Asia	Single or multiple nodules or cavities	

The diagnosis of an *Aspergillus* nodule may be challenging. Almost one third of patients did not have a positive *Aspergillus* IgG, and only 12 % had detectable *Aspergillus fumigatus* precipitins. Additionally the clinical features may be non-specific, and similar to those in patients presenting with malignant disease. In this study, cough alone was the most common clinical finding. The demographics of the patients diagnosed with *Aspergillus* nodules are also similar to those diagnosed with malignant conditions of the lung. Our centre previously reported on the PET imaging in patients with CPA [17]. In that series all of the patients had positive PET imaging. Only a small number of patients in this current series had PET scans available for review, but all eight were positive with low-moderate FDG uptake.

This study is limited by being a retrospective review. Case finding was challenging, and despite a number of sources being utilised to identify cases, it is possible some cases were missed. However, this is the largest series of *Aspergillus* nodules published to date, and the only study to correlate radiology and histology findings with clinical features and laboratory parameters, in particular *Aspergillus* IgG. In patients with chronic cavitary pulmonary aspergillosis, we found the ImmunoCap Aspergillus IgG assay to be 96 % sensitive and 98 % specific at a cutoff of 20 mg/L and 88 % sensitive and 100 % specific at the current manufacturer’s cutoff of 40 mg/L, compared to a healthy younger control population [15]. So 69 to 81 % of patients in this series had positive *Aspergillus* IgG serology, depending on the cutoff used [18]. This further highlights this previously lesser recognised manifestation of CPA.

**Fig. 4:**
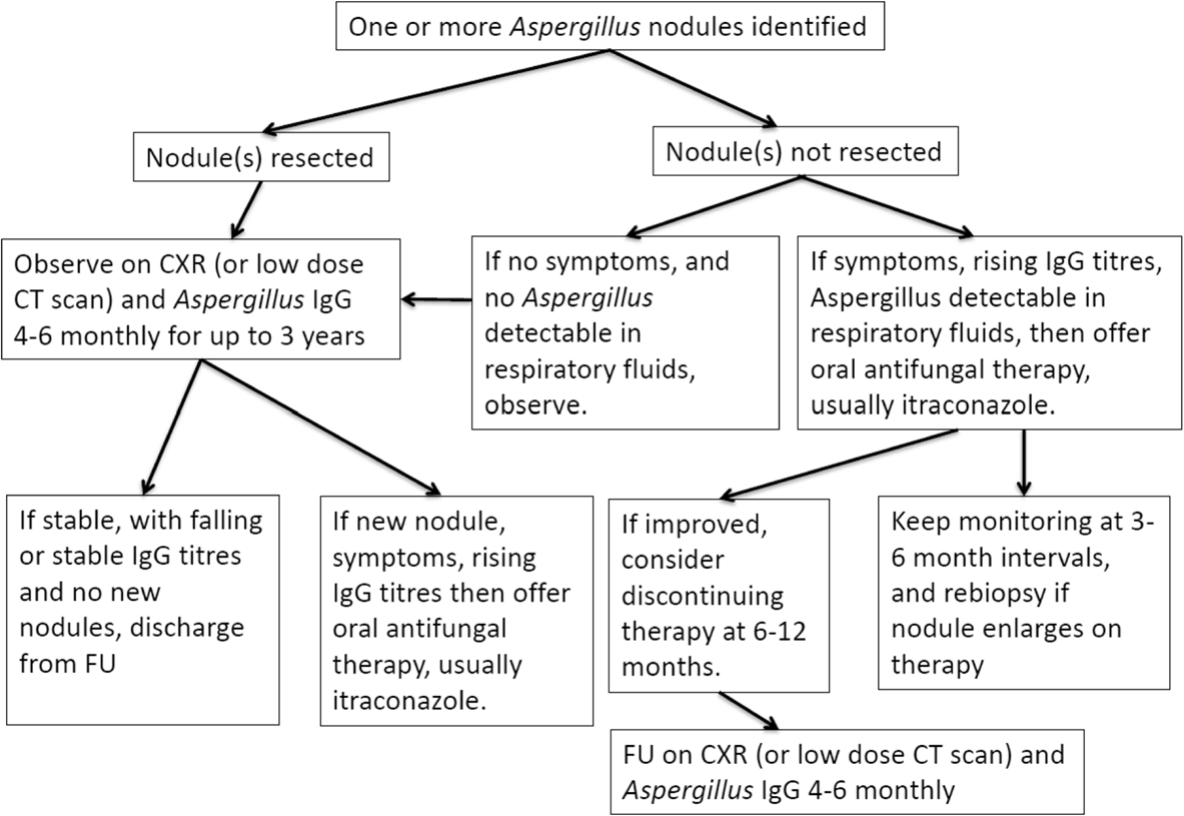
Current management algorithm for *Aspergillus* nodules. CXR = chest radiograph; CT = computed tomography; FU = follow up

The natural history of an *Aspergillus* nodule is not known. We are unable to define how long they were present before they came to medical attention, but we suspect months. We do know that some of the nodules remain stable off therapy for months or years after diagnosis. In general we treated the symptomatic patients, especially those with multiple lesions. Detection of *Aspergillus* in airways with culture or PCR also influenced us to treat, especially in those with difficult to control asthma or ABPA. We will report long term outcomes in a subsequent paper. We summarise our current approach to management in Fig. 4.

## Conclusion

Pulmonary nodules are a less frequent manifestation of chronic pulmonary aspergillosis in immune competent patients. The natural history of these nodules is not yet defined. In this series, cough alone was a common presenting symptom. It may be difficult to distinguish Aspergillus nodules from other pathology on CT findings alone, and PET imaging would seem to be non discriminatory. Additionally, a significant proportion of these patients do not have a detectable Aspergillus IgG, meaning biopsy is necessary to exclude malignant disease. However, chronic pulmonary aspergillosis, should be a differential diagnosis in patients presenting with single or multiple pulmonary nodules.

## Abbreviations

BAL, bronchoalveolar lavage; CCPA, chronic cavitary pulmonary aspergillosis; CFPA, chronic fibrosing pulmonary aspergillosis; CNPA, chronic necrotising pulmonary aspergillosis; CPA, chronic pulmonary aspergillosis; CT, computer tomography; FDG, fluorodeoxyglucose; NAC, National Aspergillosis Centre; PET, positron emission tomography; SAIA, subacute invasive pulmonary aspergillosis; TB, tuberculosis
